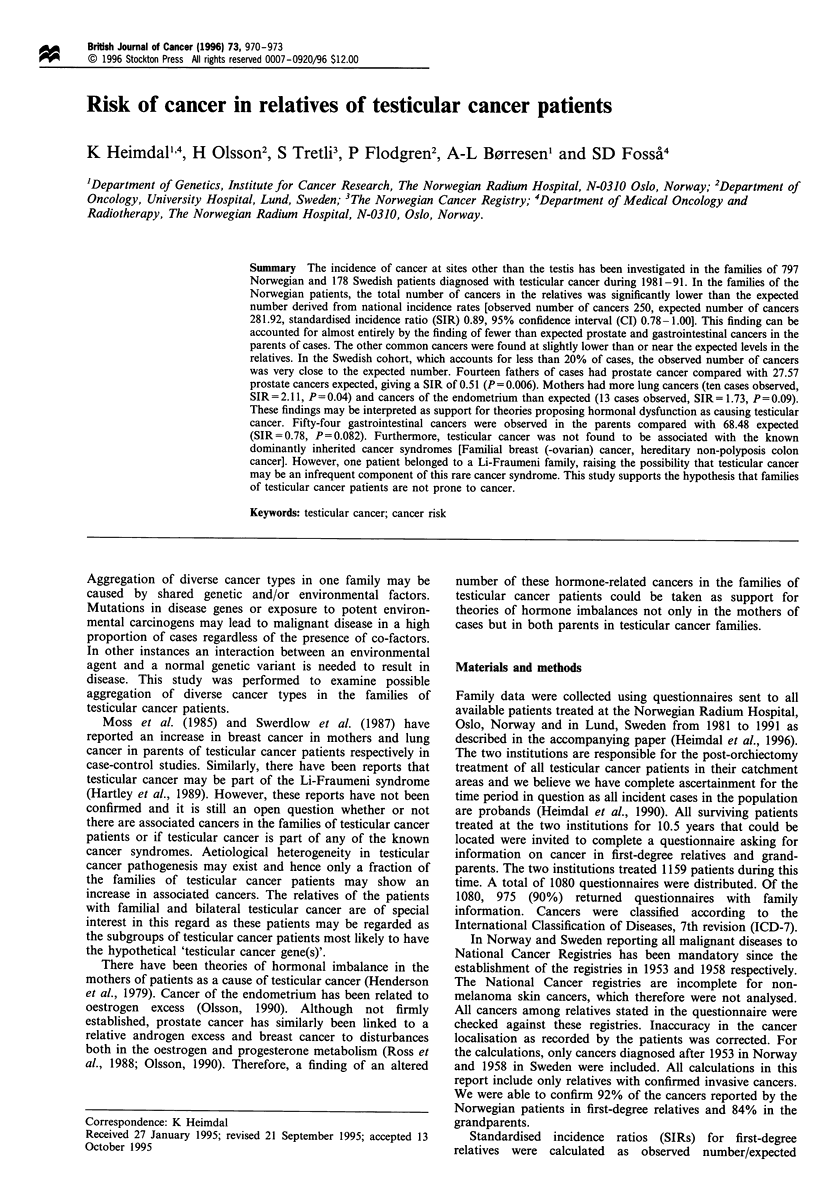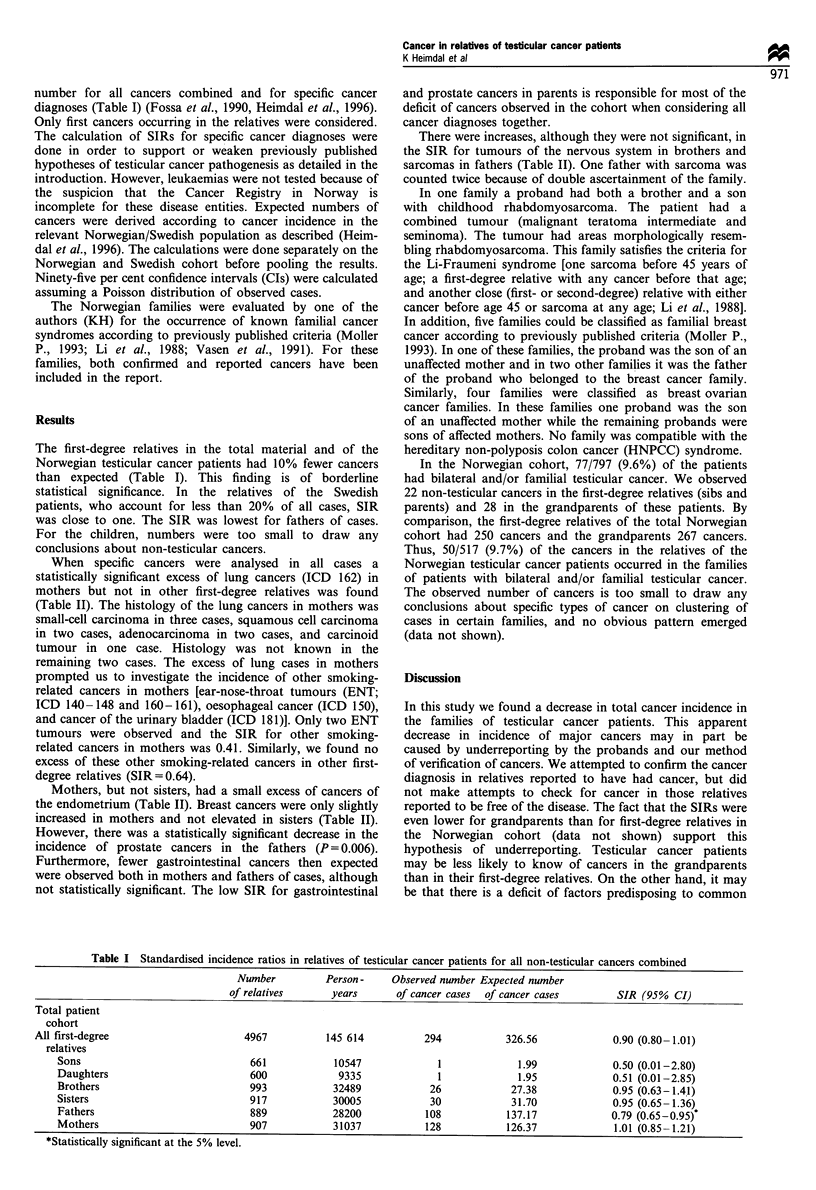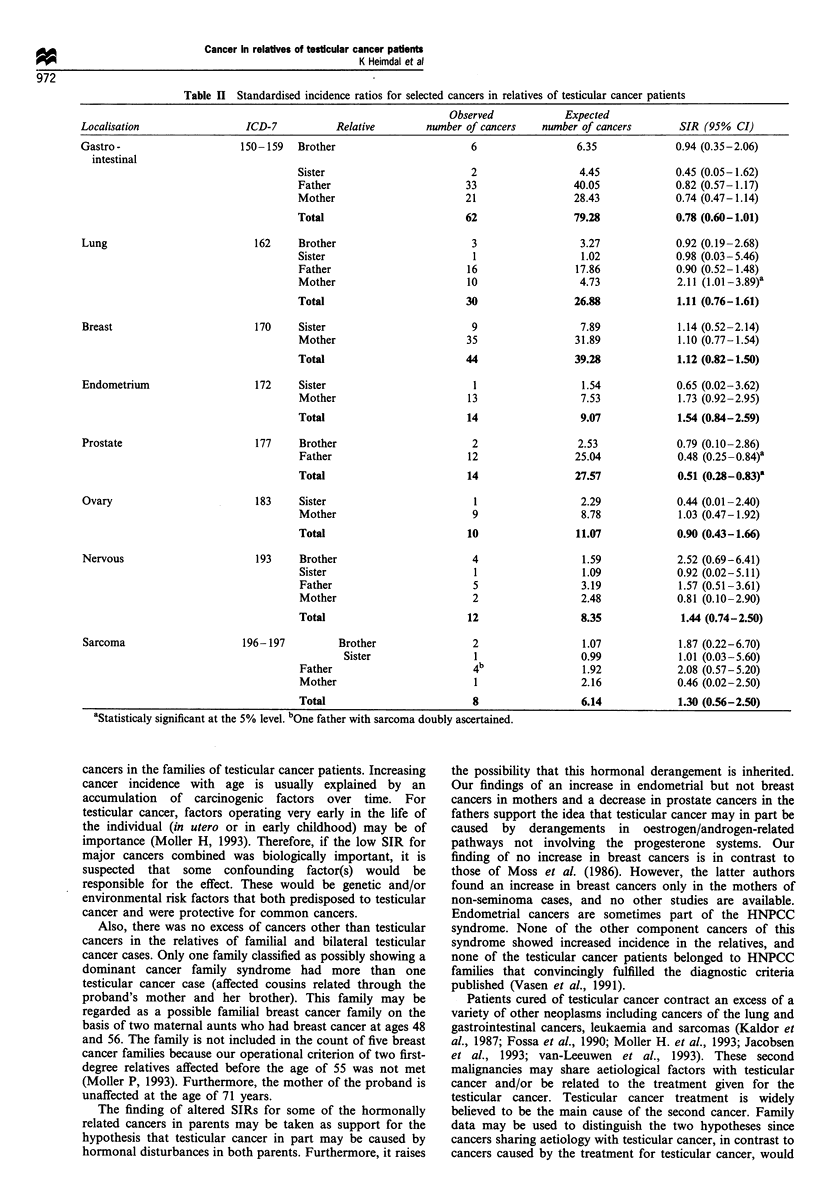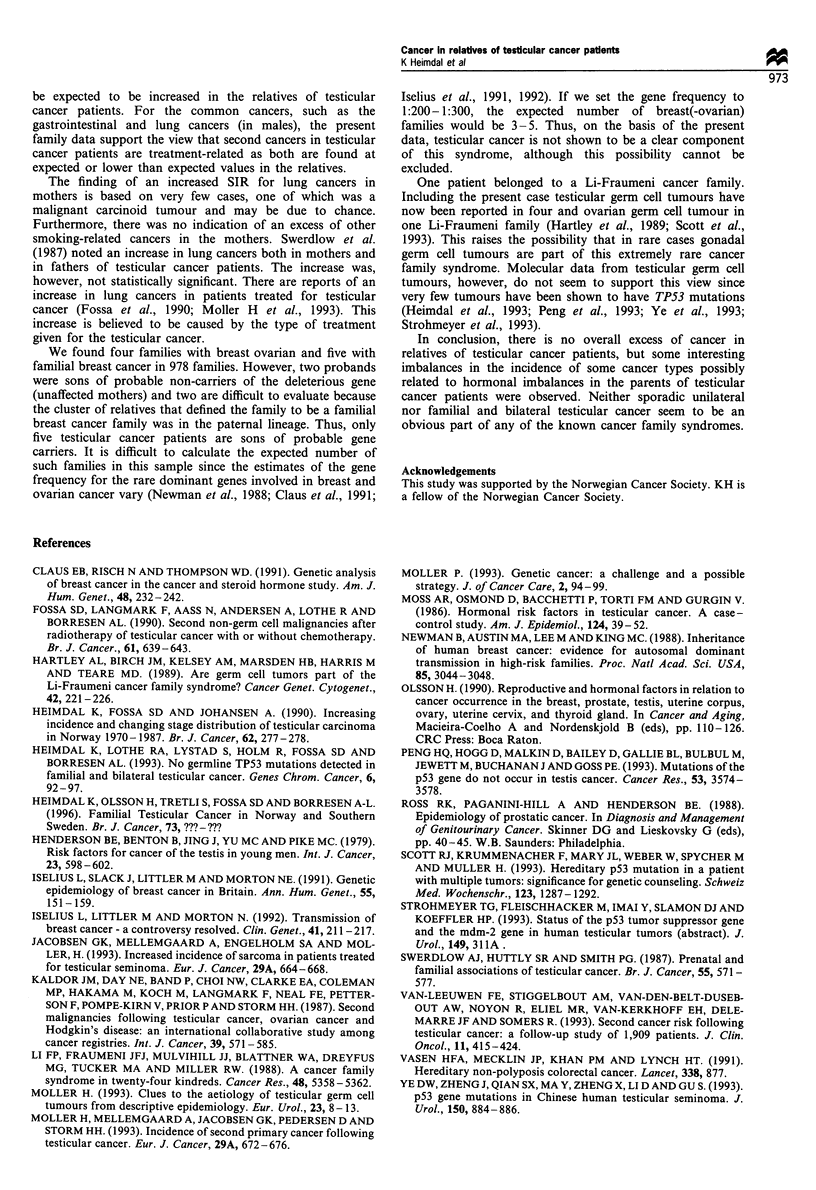# Risk of cancer in relatives of testicular cancer patients.

**DOI:** 10.1038/bjc.1996.174

**Published:** 1996-04

**Authors:** K. Heimdal, H. Olsson, S. Tretli, P. Flodgren, A. L. Børresen, S. D. Fossa

**Affiliations:** Department of Genetics, Institute for Cancer Research, The Norwegian Radium Hospital, Oslo, Norway.

## Abstract

The incidence of cancer at sites other than the testis has been investigated in the families of 797 Norwegian and 178 Swedish patients diagnosed with testicular cancer during 1981-91. In the families of the Norwegian patients, the total number of cancers in the relatives was significantly lower than the expected number derived from national incidence rates [observed number of cancers 250, expected number of cancers 281.92, standardised incidence ratio (SIR) 0.89, 95% confidence interval (CI) 0.78-1.00]. This finding can be accounted for almost entirely by the finding of fewer than expected prostate and gastrointestinal cancers in the parents of cases. The other common cancers were found at slightly lower than or near the expected levels in the relatives. In the Swedish cohort, which accounts for less than 20% of cases, the observed number of cancers was very close to the expected number. Fourteen fathers of cases had prostate cancer compared with 27.57 prostate cancers expected, giving a SIR of 0.51 (P=0.006). Mothers had more lung cancers (ten cases observed, SIR=2.11, P=0.04) and cancers of the endometrium than expected (13 cases observed, SIR=1.73, P=0.09). These findings may be interpreted as support for theories proposing hormonal dysfunction as causing testicular cancer. Fifty-four gastrointestinal cancers were observed in the parents compared with 68.48 expected (SIR=0.78, P=0.082). Furthermore, testicular cancer was not found to be associated with the known dominantly inherited cancer syndromes [Familial breast (-ovarian) cancer, hereditary no-polyposis colon cancer]. However, one patient belonged to a Li-Fraumeni family, raising the possibility that testicular cancer may be an infrequent component of this rare cancer syndrome. This study supports the hypothesis that families of testicular cancer patients are not prone to cancer.


British Journal of Cancer (1996) 73, 970-973
© 1996 Stockton Press All rights reserved 0007-0920/96 $12.00

### Risk of cancer in relatives of testicular cancer patients

K Heimdal[1,4], H Olsson[2], S Tretli[3], P Flodgren[2], A-L Børresen[1] and SD Fosså[4]

[1]*Department of Genetics, Institute for Cancer Research, The Norwegian Radium Hospital, N-0310 Oslo, Norway;* [2]*Department of Oncology, University Hospital, Lund, Sweden;* [3]*The Norwegian Cancer Registry;* [4]*Department of Medical Oncology and Radiotherapy, The Norwegian Radium Hospital, N-0310, Oslo, Norway.*

**Summary** The incidence of cancer at sites other than the testis has been investigated in the families of 797 Norwegian and 178 Swedish patients diagnosed with testicular cancer during 1981–91. In the families of the Norwegian patients, the total number of cancers in the relatives was significantly lower than the expected number derived from national incidence rates [observed number of cancers 250, expected number of cancers 281.92, standardised incidence ratio (SIR) 0.89, 95% confidence interval (CI) 0.78–1.00]. This finding can be accounted for almost entirely by the finding of fewer than expected prostate and gastrointestinal cancers in the parents of cases. The other common cancers were found at slightly lower than or near the expected levels in the relatives. In the Swedish cohort, which accounts for less than 20% of cases, the observed number of cancers was very close to the expected number. Fourteen fathers of cases had prostate cancer compared with 27.57 prostate cancers expected, giving a SIR of 0.51 ($P=0.006$). Mothers had more lung cancers (ten cases observed, SIR=2.11, $P=0.04$) and cancers of the endometrium than expected (13 cases observed, SIR=1.73, $P=0.09$). These findings may be interpreted as support for theories proposing hormonal dysfunction as causing testicular cancer. Fifty-four gastrointestinal cancers were observed in the parents compared with 68.48 expected (SIR=0.78, $P=0.082$). Furthermore, testicular cancer was not found to be associated with the known dominantly inherited cancer syndromes [Familial breast (-ovarian) cancer, hereditary non-polyposis colon cancer]. However, one patient belonged to a Li-Fraumeni family, raising the possibility that testicular cancer may be an infrequent component of this rare cancer syndrome. This study supports the hypothesis that families of testicular cancer patients are not prone to cancer.

**Keywords:** testicular cancer; cancer risk

Aggregation of diverse cancer types in one family may be caused by shared genetic and/or environmental factors. Mutations in disease genes or exposure to potent environmental carcinogens may lead to malignant disease in a high proportion of cases regardless of the presence of co-factors. In other instances an interaction between an environmental agent and a normal genetic variant is needed to result in disease. This study was performed to examine possible aggregation of diverse cancer types in the families of testicular cancer patients.

Moss *et al.* (1985) and Swerdlow *et al.* (1987) have reported an increase in breast cancer in mothers and lung cancer in parents of testicular cancer patients respectively in case-control studies. Similarly, there have been reports that testicular cancer may be part of the Li-Fraumeni syndrome (Hartley *et al.*, 1989). However, these reports have not been confirmed and it is still an open question whether or not there are associated cancers in the families of testicular cancer patients or if testicular cancer is part of any of the known cancer syndromes. Aetiological heterogeneity in testicular cancer pathogenesis may exist and hence only a fraction of the families of testicular cancer patients may show an increase in associated cancers. The relatives of the patients with familial and bilateral testicular cancer are of special interest in this regard as these patients may be regarded as the subgroups of testicular cancer patients most likely to have the hypothetical 'testicular cancer gene(s)'.

There have been theories of hormonal imbalance in the mothers of patients as a cause of testicular cancer (Henderson *et al.*, 1979). Cancer of the endometrium has been related to oestrogen excess (Olsson, 1990). Although not firmly established, prostate cancer has similarly been linked to a relative androgen excess and breast cancer to disturbances both in the oestrogen and progesterone metabolism (Ross *et al.*, 1988; Olsson, 1990). Therefore, a finding of an altered number of these hormone-related cancers in the families of testicular cancer patients could be taken as support for theories of hormone imbalances not only in the mothers of cases but in both parents in testicular cancer families.

Correspondence: K Heimdal
Received 27 January 1995; revised 21 September 1995; accepted 13 October 1995

### Materials and methods

Family data were collected using questionnaires sent to all available patients treated at the Norwegian Radium Hospital, Oslo, Norway and in Lund, Sweden from 1981 to 1991 as described in the accompanying paper (Heimdal *et al.*, 1996). The two institutions are responsible for the post-orchiectomy treatment of all testicular cancer patients in their catchment areas and we believe we have complete ascertainment for the time period in question as all incident cases in the population are probands (Heimdal *et al.*, 1990). All surviving patients treated at the two institutions for 10.5 years that could be located were invited to complete a questionnaire asking for information on cancer in first-degree relatives and grandparents. The two institutions treated 1159 patients during this time. A total of 1080 questionnaires were distributed. Of the 1080, 975 (90%) returned questionnaires with family information. Cancers were classified according to the International Classification of Diseases, 7th revision (ICD-7).

In Norway and Sweden reporting all malignant diseases to National Cancer Registries has been mandatory since the establishment of the registries in 1953 and 1958 respectively. The National Cancer registries are incomplete for non-melanoma skin cancers, which therefore were not analysed. All cancers among relatives stated in the questionnaire were checked against these registries. Inaccuracy in the cancer localisation as recorded by the patients was corrected. For the calculations, only cancers diagnosed after 1953 in Norway and 1958 in Sweden were included. All calculations in this report include only relatives with confirmed invasive cancers. We were able to confirm 92% of the cancers reported by the Norwegian patients in first-degree relatives and 84% in the grandparents.

Standardised incidence ratios (SIRs) for first-degree relatives were calculated as observed number/expected number for all cancers combined and for specific cancer diagnoses (Table I) (Fossa et al., 1990, Heimdal et al., 1996). Only first cancers occurring in the relatives were considered. The calculation of SIRs for specific cancer diagnoses were done in order to support or weaken previously published hypotheses of testicular cancer pathogenesis as detailed in the introduction. However, leukaemias were not tested because of the suspicion that the Cancer Registry in Norway is incomplete for these disease entities. Expected numbers of cancers were derived according to cancer incidence in the relevant Norwegian/Swedish population as described (Heimdal et al., 1996). The calculations were done separately on the Norwegian and Swedish cohort before pooling the results. Ninety-five per cent confidence intervals (CIs) were calculated assuming a Poisson distribution of observed cases.

The Norwegian families were evaluated by one of the authors (KH) for the occurrence of known familial cancer syndromes according to previously published criteria (Moller P., 1993; Li et al., 1988; Vasen et al., 1991). For these families, both confirmed and reported cancers have been included in the report.

### Results

The first-degree relatives in the total material and of the Norwegian testicular cancer patients had 10% fewer cancers than expected (Table I). This finding is of borderline statistical significance. In the relatives of the Swedish patients, who account for less than 20% of all cases, SIR was close to one. The SIR was lowest for fathers of cases. For the children, numbers were too small to draw any conclusions about non-testicular cancers.

When specific cancers were analysed in all cases a statistically significant excess of lung cancers (ICD 162) in mothers but not in other first-degree relatives was found (Table II). The histology of the lung cancers in mothers was small-cell carcinoma in three cases, squamous cell carcinoma in two cases, adenocarcinoma in two cases, and carcinoid tumour in one case. Histology was not known in the remaining two cases. The excess of lung cases in mothers prompted us to investigate the incidence of other smoking-related cancers in mothers [ear-nose-throat tumours (ENT; ICD 140–148 and 160–161), oesophageal cancer (ICD 150), and cancer of the urinary bladder (ICD 181)]. Only two ENT tumours were observed and the SIR for other smoking-related cancers in mothers was 0.41. Similarly, we found no excess of these other smoking-related cancers in other first-degree relatives (SIR = 0.64).

Mothers, but not sisters, had a small excess of cancers of the endometrium (Table II). Breast cancers were only slightly increased in mothers and not elevated in sisters (Table II). However, there was a statistically significant decrease in the incidence of prostate cancers in the fathers ($P = 0.006$). Furthermore, fewer gastrointestinal cancers then expected were observed both in mothers and fathers of cases, although not statistically significant. The low SIR for gastrointestinal and prostate cancers in parents is responsible for most of the deficit of cancers observed in the cohort when considering all cancer diagnoses together.

There were increases, although they were not significant, in the SIR for tumours of the nervous system in brothers and sarcomas in fathers (Table II). One father with sarcoma was counted twice because of double ascertainment of the family.

In one family a proband had both a brother and a son with childhood rhabdomyosarcoma. The patient had a combined tumour (malignant teratoma intermediate and seminoma). The tumour had areas morphologically resembling rhabdomyosarcoma. This family satisfies the criteria for the Li-Fraumeni syndrome [one sarcoma before 45 years of age; a first-degree relative with any cancer before that age; and another close (first- or second-degree) relative with either cancer before age 45 or sarcoma at any age; Li et al., 1988]. In addition, five families could be classified as familial breast cancer according to previously published criteria (Moller P., 1993). In one of these families, the proband was the son of an unaffected mother and in two other families it was the father of the proband who belonged to the breast cancer family. Similarly, four families were classified as breast ovarian cancer families. In these families one proband was the son of an unaffected mother while the remaining probands were sons of affected mothers. No family was compatible with the hereditary non-polyposis colon cancer (HNPCC) syndrome.

In the Norwegian cohort, 77/797 (9.6%) of the patients had bilateral and/or familial testicular cancer. We observed 22 non-testicular cancers in the first-degree relatives (sibs and parents) and 28 in the grandparents of these patients. By comparison, the first-degree relatives of the total Norwegian cohort had 250 cancers and the grandparents 267 cancers. Thus, 50/517 (9.7%) of the cancers in the relatives of the Norwegian testicular cancer patients occurred in the families of patients with bilateral and/or familial testicular cancer. The observed number of cancers is too small to draw any conclusions about specific types of cancer on clustering of cases in certain families, and no obvious pattern emerged (data not shown).

### Discussion

In this study we found a decrease in total cancer incidence in the families of testicular cancer patients. This apparent decrease in incidence of major cancers may in part be caused by underreporting by the probands and our method of verification of cancers. We attempted to confirm the cancer diagnosis in relatives reported to have had cancer, but did not make attempts to check for cancer in those relatives reported to be free of the disease. The fact that the SIRs were even lower for grandparents than for first-degree relatives in the Norwegian cohort (data not shown) support this hypothesis of underreporting. Testicular cancer patients may be less likely to know of cancers in the grandparents than in their first-degree relatives. On the other hand, it may be that there is a deficit of factors predisposing to common

**Table I** Standardised incidence ratios in relatives of testicular cancer patients for all non-testicular cancers combined

| | *Number of relatives* | *Person-years* | *Observed number of cancer cases* | *Expected number of cancer cases* | *SIR (95% CI)* |
|---|---|---|---|---|---|
| Total patient cohort | | | | | |
| All first-degree relatives | 4967 | 145 614 | 294 | 326.56 | 0.90 (0.80–1.01) |
| Sons | 661 | 10547 | 1 | 1.99 | 0.50 (0.01–2.80) |
| Daughters | 600 | 9335 | 1 | 1.95 | 0.51 (0.01–2.85) |
| Brothers | 993 | 32489 | 26 | 27.38 | 0.95 (0.63–1.41) |
| Sisters | 917 | 30005 | 30 | 31.70 | 0.95 (0.65–1.36) |
| Fathers | 889 | 28200 | 108 | 137.17 | 0.79 (0.65–0.95)* |
| Mothers | 907 | 31037 | 128 | 126.37 | 1.01 (0.85–1.21) |

*Statistically significant at the 5% level.

Table II Standardised incidence ratios for selected cancers in relatives of testicular cancer patients

| Localisation | ICD-7 | Relative | Observed number of cancers | Expected number of cancers | SIR (95% CI) |
|---|---|---|---|---|---|
| Gastro-intestinal | 150–159 | Brother | 6 | 6.35 | 0.94 (0.35–2.06) |
| | | Sister | 2 | 4.45 | 0.45 (0.05–1.62) |
| | | Father | 33 | 40.05 | 0.82 (0.57–1.17) |
| | | Mother | 21 | 28.43 | 0.74 (0.47–1.14) |
| | | **Total** | **62** | **79.28** | **0.78 (0.60–1.01)** |
| Lung | 162 | Brother | 3 | 3.27 | 0.92 (0.19–2.68) |
| | | Sister | 1 | 1.02 | 0.98 (0.03–5.46) |
| | | Father | 16 | 17.86 | 0.90 (0.52–1.48) |
| | | Mother | 10 | 4.73 | 2.11 (1.01–3.89)[a] |
| | | **Total** | **30** | **26.88** | **1.11 (0.76–1.61)** |
| Breast | 170 | Sister | 9 | 7.89 | 1.14 (0.52–2.14) |
| | | Mother | 35 | 31.89 | 1.10 (0.77–1.54) |
| | | **Total** | **44** | **39.28** | **1.12 (0.82–1.50)** |
| Endometrium | 172 | Sister | 1 | 1.54 | 0.65 (0.02–3.62) |
| | | Mother | 13 | 7.53 | 1.73 (0.92–2.95) |
| | | **Total** | **14** | **9.07** | **1.54 (0.84–2.59)** |
| Prostate | 177 | Brother | 2 | 2.53 | 0.79 (0.10–2.86) |
| | | Father | 12 | 25.04 | 0.48 (0.25–0.84)[a] |
| | | **Total** | **14** | **27.57** | **0.51 (0.28–0.83)[a]** |
| Ovary | 183 | Sister | 1 | 2.29 | 0.44 (0.01–2.40) |
| | | Mother | 9 | 8.78 | 1.03 (0.47–1.92) |
| | | **Total** | **10** | **11.07** | **0.90 (0.43–1.66)** |
| Nervous | 193 | Brother | 4 | 1.59 | 2.52 (0.69–6.41) |
| | | Sister | 1 | 1.09 | 0.92 (0.02–5.11) |
| | | Father | 5 | 3.19 | 1.57 (0.51–3.61) |
| | | Mother | 2 | 2.48 | 0.81 (0.10–2.90) |
| | | **Total** | **12** | **8.35** | **1.44 (0.74–2.50)** |
| Sarcoma | 196–197 | Brother | 2 | 1.07 | 1.87 (0.22–6.70) |
| | | Sister | 1 | 0.99 | 1.01 (0.03–5.60) |
| | | Father | 4[b] | 1.92 | 2.08 (0.57–5.20) |
| | | Mother | 1 | 2.16 | 0.46 (0.02–2.50) |
| | | **Total** | **8** | **6.14** | **1.30 (0.56–2.50)** |

[a]Statisticaly significant at the 5% level. [b]One father with sarcoma doubly ascertained.

cancers in the families of testicular cancer patients. Increasing cancer incidence with age is usually explained by an accumulation of carcinogenic factors over time. For testicular cancer, factors operating very early in the life of the individual (*in utero* or in early childhood) may be of importance (Moller H, 1993). Therefore, if the low SIR for major cancers combined was biologically important, it is suspected that some confounding factor(s) would be responsible for the effect. These would be genetic and/or environmental risk factors that both predisposed to testicular cancer and were protective for common cancers.

Also, there was no excess of cancers other than testicular cancers in the relatives of familial and bilateral testicular cancer cases. Only one family classified as possibly showing a dominant cancer family syndrome had more than one testicular cancer case (affected cousins related through the proband's mother and her brother). This family may be regarded as a possible familial breast cancer family on the basis of two maternal aunts who had breast cancer at ages 48 and 56. The family is not included in the count of five breast cancer families because our operational criterion of two first-degree relatives affected before the age of 55 was not met (Moller P, 1993). Furthermore, the mother of the proband is unaffected at the age of 71 years.

The finding of altered SIRs for some of the hormonally related cancers in parents may be taken as support for the hypothesis that testicular cancer in part may be caused by hormonal disturbances in both parents. Furthermore, it raises the possibility that this hormonal derangement is inherited. Our findings of an increase in endometrial but not breast cancers in mothers and a decrease in prostate cancers in the fathers support the idea that testicular cancer may in part be caused by derangements in oestrogen/androgen-related pathways not involving the progesterone systems. Our finding of no increase in breast cancers is in contrast to those of Moss *et al.* (1986). However, the latter authors found an increase in breast cancers only in the mothers of non-seminoma cases, and no other studies are available. Endometrial cancers are sometimes part of the HNPCC syndrome. None of the other component cancers of this syndrome showed increased incidence in the relatives, and none of the testicular cancer patients belonged to HNPCC families that convincingly fulfilled the diagnostic criteria published (Vasen *et al.*, 1991).

Patients cured of testicular cancer contract an excess of a variety of other neoplasms including cancers of the lung and gastrointestinal cancers, leukaemia and sarcomas (Kaldor *et al.*, 1987; Fossa *et al.*, 1990; Moller H. *et al.*, 1993; Jacobsen *et al.*, 1993; van-Leeuwen *et al.*, 1993). These second malignancies may share aetiological factors with testicular cancer and/or be related to the treatment given for the testicular cancer. Testicular cancer treatment is widely believed to be the main cause of the second cancer. Family data may be used to distinguish the two hypotheses since cancers sharing aetiology with testicular cancer, in contrast to cancers caused by the treatment for testicular cancer, would be expected to be increased in the relatives of testicular cancer patients. For the common cancers, such as the gastrointestinal and lung cancers (in males), the present family data support the view that second cancers in testicular cancer patients are treatment-related as both are found at expected or lower than expected values in the relatives.

The finding of an increased SIR for lung cancers in mothers is based on very few cases, one of which was a malignant carcinoid tumour and may be due to chance. Furthermore, there was no indication of an excess of other smoking-related cancers in the mothers. Swerdlow et al. (1987) noted an increase in lung cancers both in mothers and in fathers of testicular cancer patients. The increase was, however, not statistically significant. There are reports of an increase in lung cancers in patients treated for testicular cancer (Fossa et al., 1990; Moller H et al., 1993). This increase is believed to be caused by the type of treatment given for the testicular cancer.

We found four families with breast ovarian and five with familial breast cancer in 978 families. However, two probands were sons of probable non-carriers of the deleterious gene (unaffected mothers) and two are difficult to evaluate because the cluster of relatives that defined the family to be a familial breast cancer family was in the paternal lineage. Thus, only five testicular cancer patients are sons of probable gene carriers. It is difficult to calculate the expected number of such families in this sample since the estimates of the gene frequency for the rare dominant genes involved in breast and ovarian cancer vary (Newman et al., 1988; Claus et al., 1991; Iselius et al., 1991, 1992). If we set the gene frequency to 1:200–1:300, the expected number of breast(-ovarian) families would be 3–5. Thus, on the basis of the present data, testicular cancer is not shown to be a clear component of this syndrome, although this possibility cannot be excluded.

One patient belonged to a Li-Fraumeni cancer family. Including the present case testicular germ cell tumours have now been reported in four and ovarian germ cell tumour in one Li-Fraumeni family (Hartley et al., 1989; Scott et al., 1993). This raises the possibility that in rare cases gonadal germ cell tumours are part of this extremely rare cancer family syndrome. Molecular data from testicular germ cell tumours, however, do not seem to support this view since very few tumours have been shown to have *TP53* mutations (Heimdal et al., 1993; Peng et al., 1993; Ye et al., 1993; Strohmeyer et al., 1993).

In conclusion, there is no overall excess of cancer in relatives of testicular cancer patients, but some interesting imbalances in the incidence of some cancer types possibly related to hormonal imbalances in the parents of testicular cancer patients were observed. Neither sporadic unilateral nor familial and bilateral testicular cancer seem to be an obvious part of any of the known cancer family syndromes.

**Acknowledgements**
This study was supported by the Norwegian Cancer Society. KH is a fellow of the Norwegian Cancer Society.

### References

CLAUS EB, RISCH N AND THOMPSON WD. (1991). Genetic analysis of breast cancer in the cancer and steroid hormone study. *Am. J. Hum. Genet.*, **48**, 232–242.

FOSSA SD, LANGMARK F, AASS N, ANDERSEN A, LOTHE R AND BORRESEN AL. (1990). Second non-germ cell malignancies after radiotherapy of testicular cancer with or without chemotherapy. *Br. J. Cancer.*, **61**, 639–643.

HARTLEY AL, BIRCH JM, KELSEY AM, MARSDEN HB, HARRIS M AND TEARE MD. (1989). Are germ cell tumors part of the Li-Fraumeni cancer family syndrome? *Cancer Genet. Cytogenet.*, **42**, 221–226.

HEIMDAL K, FOSSA SD AND JOHANSEN A. (1990). Increasing incidence and changing stage distribution of testicular carcinoma in Norway 1970–1987. *Br. J. Cancer*, **62**, 277–278.

HEIMDAL K, LOTHE RA, LYSTAD S, HOLM R, FOSSA SD AND BORRESEN AL. (1993). No germline TP53 mutations detected in familial and bilateral testicular cancer. *Genes Chrom. Cancer*, **6**, 92–97.

HEIMDAL K, OLSSON H, TRETLI S, FOSSA SD AND BORRESEN A-L. (1996). Familial Testicular Cancer in Norway and Southern Sweden. *Br. J. Cancer*, **73**, ???–???

HENDERSON BE, BENTON B, JING J, YU MC AND PIKE MC. (1979). Risk factors for cancer of the testis in young men. *Int. J. Cancer*, **23**, 598–602.

ISELIUS L, SLACK J, LITTLER M AND MORTON NE. (1991). Genetic epidemiology of breast cancer in Britain. *Ann. Hum. Genet.*, **55**, 151–159.

ISELIUS L, LITTLER M AND MORTON N. (1992). Transmission of breast cancer - a controversy resolved. *Clin. Genet.*, **41**, 211–217.

JACOBSEN GK, MELLEMGAARD A, ENGELHOLM SA AND MOLLER, H. (1993). Increased incidence of sarcoma in patients treated for testicular seminoma. *Eur. J. Cancer*, **29A**, 664–668.

KALDOR JM, DAY NE, BAND P, CHOI NW, CLARKE EA, COLEMAN MP, HAKAMA M, KOCH M, LANGMARK F, NEAL FE, PETTERSON F, POMPE-KIRN V, PRIOR P AND STORM HH. (1987). Second malignancies following testicular cancer, ovarian cancer and Hodgkin's disease: an international collaborative study among cancer registries. *Int. J. Cancer*, **39**, 571–585.

LI FP, FRAUMENI JFJ, MULVIHILL JJ, BLATTNER WA, DREYFUS MG, TUCKER MA AND MILLER RW. (1988). A cancer family syndrome in twenty-four kindreds. *Cancer Res.*, **48**, 5358–5362.

MOLLER H. (1993). Clues to the aetiology of testicular germ cell tumours from descriptive epidemiology. *Eur. Urol.*, **23**, 8–13.

MOLLER H, MELLEMGAARD A, JACOBSEN GK, PEDERSEN D AND STORM HH. (1993). Incidence of second primary cancer following testicular cancer. *Eur. J. Cancer*, **29A**, 672–676.

MOLLER P. (1993). Genetic cancer: a challenge and a possible strategy. *J. of Cancer Care*, **2**, 94–99.

MOSS AR, OSMOND D, BACCHETTI P, TORTI FM AND GURGIN V. (1986). Hormonal risk factors in testicular cancer. A case–control study. *Am. J. Epidemiol.*, **124**, 39–52.

NEWMAN B, AUSTIN MA, LEE M AND KING MC. (1988). Inheritance of human breast cancer: evidence for autosomal dominant transmission in high-risk families. *Proc. Natl Acad. Sci. USA*, **85**, 3044–3048.

OLSSON H. (1990). Reproductive and hormonal factors in relation to cancer occurrence in the breast, prostate, testis, uterine corpus, ovary, uterine cervix, and thyroid gland. In *Cancer and Aging*, Macieira-Coelho A and Nordenskjold B (eds), pp. 110–126. CRC Press: Boca Raton.

PENG HQ, HOGG D, MALKIN D, BAILEY D, GALLIE BL, BULBUL M, JEWETT M, BUCHANAN J AND GOSS PE. (1993). Mutations of the p53 gene do not occur in testis cancer. *Cancer Res.*, **53**, 3574–3578.

ROSS RK, PAGANINI-HILL A AND HENDERSON BE. (1988). Epidemiology of prostatic cancer. In *Diagnosis and Management of Genitourinary Cancer*. Skinner DG and Lieskovsky G (eds), pp. 40–45. W.B. Saunders: Philadelphia.

SCOTT RJ, KRUMMENACHER F, MARY JL, WEBER W, SPYCHER M AND MULLER H. (1993). Hereditary p53 mutation in a patient with multiple tumors: significance for genetic counseling. *Schweiz Med. Wochenschr.*, **123**, 1287–1292.

STROHMEYER TG, FLEISCHHACKER M, IMAI Y, SLAMON DJ AND KOEFFLER HP. (1993). Status of the p53 tumor suppressor gene and the mdm-2 gene in human testicular tumors (abstract). *J. Urol.*, **149**, 311A .

SWERDLOW AJ, HUTTLY SR AND SMITH PG. (1987). Prenatal and familial associations of testicular cancer. *Br. J. Cancer*, **55**, 571–577.

VAN-LEEUWEN FE, STIGGELBOUT AM, VAN-DEN-BELT-DUSEBOUT AW, NOYON R, ELIEL MR, VAN-KERKHOFF EH, DELEMARRE JF AND SOMERS R. (1993). Second cancer risk following testicular cancer: a follow-up study of 1,909 patients. *J. Clin. Oncol.*, **11**, 415–424.

VASEN HFA, MECKLIN JP, KHAN PM AND LYNCH HT. (1991). Hereditary non-polyposis colorectal cancer. *Lancet*, **338**, 877.

YE DW, ZHENG J, QIAN SX, MA Y, ZHENG X, LI D AND GU S. (1993). p53 gene mutations in Chinese human testicular seminoma. *J. Urol.*, **150**, 884–886.